# Using a Partial Sum Method and GPS Tracking Data to Identify Area Restricted Search by Artisanal Fishers at Moored Fish Aggregating Devices in the Commonwealth of Dominica

**DOI:** 10.1371/journal.pone.0115552

**Published:** 2015-02-03

**Authors:** Michael Alvard, David Carlson, Ethan McGaffey

**Affiliations:** 1 Department of Anthropology, Texas A&M University, College Station, Texas, United States of America; 2 Department of Anthropology, University of North Texas, Denton, Texas, United States of America; Hokkaido University, JAPAN

## Abstract

Foragers must often travel from a central place to exploit aggregations of prey. These patches can be identified behaviorally when a forager shifts from travel to area restricted search, identified by a decrease in speed and an increase in sinuosity of movement. Faster, more directed movement is associated with travel. Differentiating foraging behavior at patches from travel to patches is important for a variety of research questions and has now been made easier by the advent of small, GPS devices that can track forager movement with high resolution. In the summer and fall of 2012, movement data were collected from GPS devices placed on foraging trips originating in the artisanal fishing village of Desa Ikan (pseudonym), on the east coast of the Caribbean island nation of the Commonwealth Dominica. Moored FADs are human-made structures anchored to the ocean floor with fish attraction material on or near the surface designed to effectively create a resource patch. The ultimate goal of the research is to understand how property rights are emerging after the introduction of fish aggregating device (FAD) technology at the site in 1999. This paper reports on research to identify area-restricted search foraging behavior at FAD patches. For 22 foraging trips simultaneous behavioral observations were made to ground-truth the GPS movement data. Using a cumulative sum method, area restricted search was identified as negative deviations from the mean travel speed and the method was able to correctly identify FAD patches in every case.

## Introduction

FADs (Fish Aggregating Devices) are manmade structures designed to float on or near the surface of a body of water, attract fish and facilitate their capture [1–3]. Moored FADs create resource patches at known locations significantly reducing search time, effort, and fuel costs for fishers. Globally, industrial drifting FADs account for an increasing proportion of many economically important fisheries [4]. In 2011, FADs accounted for over 40% of the world’s tropical tuna catches [5]. Globally widespread FAD use is a recent development. Since the 1970s the technology in various forms has spread from SE Asia where traditional FAD technology, like the *palou* of the Philippines, has been deployed by local fishers for centuries [6]. In many parts of the world, including the Caribbean, FADs are increasingly being used by small scale, artisanal fishers to access fish species otherwise difficult to harvest in large numbers [7,8]. In the Commonwealth of Dominica, a Caribbean island nation, where they have been used only since the late 1990s, FADs are now anchored in the deep water off of the insular shelf by artisanal fishers who use them seasonally to target pelagic fish, primarily tuna (*Thunnus* spp.) and marlin (*Makaira* spp.) [9].

FADs are interesting in a variety of ways. How and why they attract fish is an open question [3] and their impact on fish stocks raises concerns [10,11]. In many places, including Dominica, where legislation to regulate and management FAD fishing is still in development, FADs create property rights conflicts over who owns the aggregated fish [12,13], and the technology is so new that use-right norms are still evolving. FADs are an excellent example of niche construction [14,15] as fishers alter the environment to create patchiness in an otherwise relatively homogenous foraging seascape.

The analysis below is framed by foraging theory [16]. In the most general sense, foragers search for prey; this involves movement in space over time to facilitate encounters with patches. Patches are aggregations of prey that foragers often travel to from a central place [17]. At patch sites, like those where fish aggregate around FADs, prey encounters are expected to be spatiotemporally auto correlated [18,19]; once a forager encounters a prey item there is an increased probability of finding additional prey nearby. In this case, foraging theory predicts that foragers should conduct area-restricted search (ARS), defined by a decrease in speed and an increase in sinuosity of movement [20,21]. ARS has been identified in a wide variety of foragers, including humans [22–26]. Searching for patches differs in its nature from localized searching within a patch, sometimes referred to as extensive verses intensive search, respectively [20,27]. When traveling between patches or back to a central place the forager is expected to travel more quickly and more linearly [28].

The work that we report here is part of a larger project with the goal of understanding how Dominican artisanal FAD fishermen develop solutions (or not) to property rights conflicts. In this paper we report on research that will help us identify the resource patches created by the FADs so that we can accurately measure how different fishermen use those patches. Following the arguments above, patches will be identified behaviorally when the Dominican fishers shift from travel to ARS. Using GPS data to track the movement of the boats, we use the partial sum method developed by Knell and Codling [29,30] to identify ARS at the FADs, and test the model using simultaneous behavioral observations made to ground-truth the GPS movement data.

### Ethnographic context: Foraging trips by artisanal FAD fishermen

Data were collected between August 29 and December 15, 2012 from foraging trips originating at the medium-sized fish landing site located in the community of Desa Ikan (pseudonym) on the rural east coast of the Commonwealth of Dominica. The site features a large bay with a harbor protected behind a stone breakwater where boats are secured along the shore. The landing site is host to fishers and boat owners who live in Desa Ikan and nearby communities. Sixteen boats made 95% of all recorded trips (N = 505) out of a total of 22 boats that fished during the period reported here. Thirty-two fishers made 95% of all trips out of a total of 53 individuals who fished. Fishing at Desa Ikan is classically artisanal and small-scale [31]. Boats are small (4m–8m in length), constructed with wood or wood reinforced with fiberglass, and powered with two-stroke outboard motors that range from 15hp to 85hp motors (mode = 48hp). Modal crew size is two, and ranges from one to three persons. Trips are diurnal, rarely last more than 12 hours or travel more than 50km from shore. There is no export market in Dominica. Fish are sold locally, in nearby communities, or occasionally transported 45km by road to Roseau, the capital.

As a group, the fishers target a variety of both demersal and pelagic fish. Strategies at Desa Ikan vary with season across three general strategies. Near-shore, shallow-water, bank fishing with hook and line and traps for demersals such as snapper (*Lutjanus* spp.) is practiced year-around. Most fishers take part in the late winter-spring ‘channel’ fishing season which involves hunting for schools of dolphinfish (*Coryphaena hippurus*) and other pelagics and capturing them with hook and line in the deep water off the banks. Finally, with the introduction of FADs around 1999, a number of the fishermen have been able to target large coastal and migratory pelagic fish, particularly tuna (*Thunnus* spp.), marlin (*Makaira* spp.) and dolphinfish [32].

**Fig 1:**
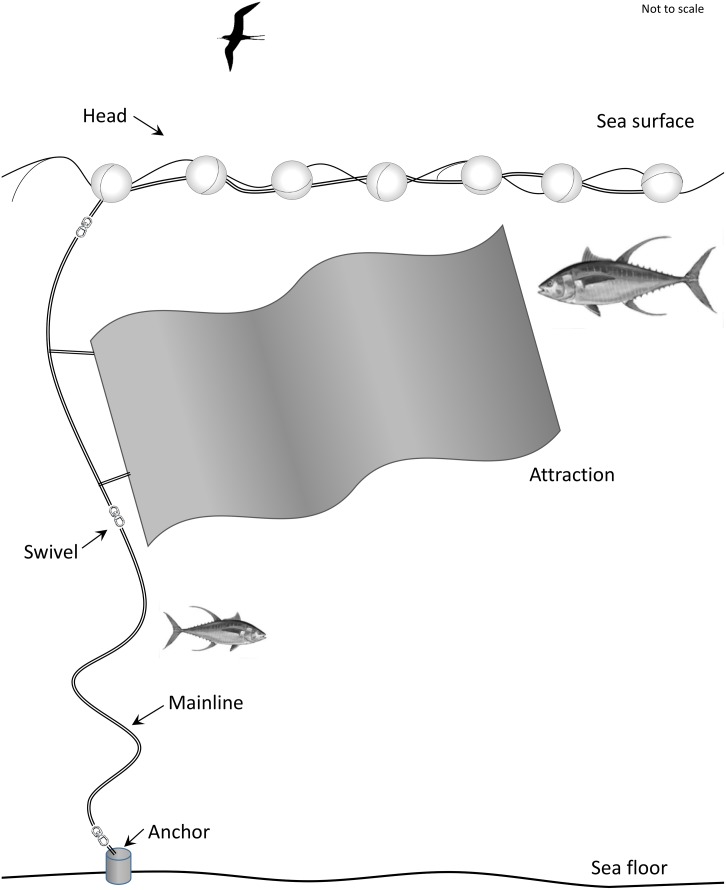
Schematic diagram of a typical FAD deployed from the field site. Designs vary around the island. The head consists of series of floats at the surface (~20cm–40cm diameter) connected by rope or other material. The head can be 20m long. Referred to as attraction in Dominica, tarps and nets are attached near the surface in order to attract fish. Attraction is sometimes attached to the head as well to the top section of mainline as shown here. Metal swivels are placed at key points to allow the device to better withstand strong current and to act as weight to keep the rope from floating to the surface. A length of polypropylene rope is attached to an anchor made from half a 55 gallon drum filled with concrete. FADs are commonly placed at depths of 1000m–2500m deep at distances of 15–50km from shore. The schematic is not to scale.

The deep water FADs deployed from Desa Ikan are anchored to the ocean floor with halved 55 gallon drums filled with concrete (Fig. 1; see also [33]). A length of polypropylene rope joins the anchor to the FAD’s head at the surface which usually consists of a number of round floats in addition to the FAD’s attraction. To prevent twisting, metal swivels are placed at key connection points. Attraction usually consists of tarps and nets attached to both the mainline near the surface and the head of the FAD. The FADs that are the focus of this research are placed in water between 1000m–2500m deep at distances of 15–50km from shore and are referred here to as deep water FADs to contrast them with the shallow water FADs that are placed close to shore and primarily target barracudas (*Sphyraena* spp).

## Materials and Methods

### Ethics Statement

This study was approved by Texas A&M University’s Office of Research Compliance Internal Review Board (Study Number: IRB2009–0209) and the Commonwealth of Dominica Fisheries Division (Research License RP-05/129 FIS-3). Texas A&M Institutional Review Board approved the use of oral consent obtained from fishers and boat owner under the criteria listed by the U. S. Code of Federal Regulations section 45 CFR 46.117. Our experience suggested that asking for signed consent in the context of ethnographic research, like that done in Dominica, puts significant strain on the research—subject relationship. It is likely that the research could not have been carried out without the waiver, as suspicion of our motives would have increased significantly. Subjects were completely informed concerning the nature of the research with the clear option of declining.

### Sample

During the data collection period, 505 fishing trips were observed leaving and returning to the landing site at Desa Ikan. We placed GPS devices aboard the boats and collected movement data from a sample of 351 of these trips [33]. For 25 of these trips, along with the GPS data, ground truth data were simultaneously collected during time-synchronized focal follows where one of us joined the crew and recorded behavioral data during the course of the trip. Twenty-three trips involved trips to FADs. One set of notes was lost overboard leaving a sample of N = 22 FAD focal follows. A variety of data were collected during these focal follows, but germane to this analysis, the observer noted when the boat was at a FAD. The analysis here is limited primarily to the 22 focal follows trips that included trips to FADs.

### GPS

The GPS loggers that were placed on the boats were model GT-31 manufactured by LOCOSYS Technology (http://www.locosystech.com/). Light and small (90mm x 58mm x 24.5mm; 98g), we housed them in hard plastic water proof cases (Pelican 1010; 196gms). The loggers use SiRF Star 3 high sensitivity, low power GPS chips energized with a built-in lithium-ion polymer rechargeable battery. Output data format is NMEA 0183, an industry standard. The manufacturer reports a 95% probability that the reported position will be within the area of a circle with a radius of 10m.around the actual location and the unobstructed conditions of the open sea were ideal for satellite reception.

The secure housing provided by the case and the compact nature of the devices cases made placement easy in the waterproof tubs that most fishers keep aboard their boats in order to keep dry a variety of their own items including their own GPS devices. Before the boats left in the morning we activated the devices and placed them in a tub or handed them to the fishers. The devices did not experience any problems receiving a signal though the two layers of plastic. We retrieved the devices when fishers returned from the sea in the afternoon. Boat owners were given a fee of 10EC ($3.7US) for each trip their boat carried a GPS device.

The GPS devices were programed to collect data at one second intervals generating a time-series with associated x, y spatial coordinates. The data have fine spatiotemporal scale and describe the movement of the boats during each fishing trip in detail. The data files can be accessed here: http://repository.tamu.edu/handle/1969.1/152005. We edited the track data in GPS Track Editor, a free software product from MapSphere (http://www.gpstrackeditor.com). Pre and post trip data points were removed from each trip’s track and the file was imported into Google Earth, a free software product from Google (http://www.google.com/earth) where they were overlaid on top of satellite imagery of the east coast of Dominica. Tracks were easily viewed across the seascape at a variety of spatial scales. Using Google Earth’s Time Slider tool the tracks could also be animated and viewed at different scales of temporal resolution.

### Ground truth via focal follows

We supplemented the GPS data collection with simultaneous behavioral observations during focal follows. For this method, a focal subject is accompanied for a period of time and data are recorded [34,35]. In this case, the subject can be considered the captain because he is responsible for directing the movement of the boat. The sample of observed trips included three captains and two boats and was not randomly selected but reflected our closer relationship with particular fishers. One boat accounted for 21 of the 22 trips; 16 of the 21 trips on that boat were with the same captain. A second individual captained for the balance of five trips on that boat. We observed one trip on a second boat that was captained by a third individual. We address issues related to the homogeneity of this sample in the discussion section below. We used event sampling to record behavioral changes or events germane to the research question and the times they occurred [36]. Salient events included departure, travel, fishing and navigation behavior, and encounters with floating debris, birds, FADs, and other boats. During the trips, our watches and the GPS devices were synchronized to allow correspondence between the subsequent GPS track and what we observed during the trips. This technique can be considered a type of ground truth common to analyses that use remotely sensed data [37]. Ground truth refers to data collected at an actual location that can be related to data collected at a distance, via satellites for example [38]. Germane to this analysis, the observer identified the time when the foragers entered and departed from foraging patches, i.e., the transition from extensive to intensive search.

#### Domain knowledge

Domain knowledge is a term from information science that refers to expert knowledge [39]. The fishers with whom we interacted are experts in the domain of FAD fishing. Discussion with fishers concerning the nature of FAD foraging before, during, and after their trips informed our conclusions based on observations; during focal follows captain and crew would confirm periods of FAD fishing. Direct observation by a research observer is also a kind of domain knowledge. Based on domain knowledge, we developed expectations regarding what tracks should look like for behavior of interest.

## Results

### Observations at the FAD

The fishermen are spatially informed foragers and enlighten analysis of the otherwise mechanistic ARS models [40]. Captains have a cognitive map generated from their memory of past foraging trips [41] as well as culturally transmitted information provided by other fishers about their experiences with FADs [42]. Captains use this information to direct their outward bound travel in the general direction of specific deep water FADs. When deployed, a FAD’s mainline is given slack (*scope*) to reduce strain in heavy current and rough seas [43] and captains know that FAD heads will drift with the ocean currents. Given the known location of the anchor drop, fishers report that the head will be located within a drift zone with a radius defined by the scope; the actual location of the head on any particular day is determined by the ocean current. Fishers who frequent FADs usually bring GPS aboard to help locate FADs. For the FADs that they regularly visit, fishers maintain coordinates of locations that lie on the circumference of drift zones and captains will search for a particular location based on their recent experience, information from others who fished the FAD recently, and conditions of wind and current. Fishers without GPS devices sometimes take advantage of those who do and follow them to FAD patches, a kind of social foraging sometimes seen in nonhuman foragers [44]. If a FAD has not been visited in some time, or if few marks are known, more scouting may be required. If the current is strong, a FAD head can be pulled under the surface and cannot be found.

Based on experience, captains have an approximate idea how much time it will take them to travel to the area where the target FAD is located. If there is a GPS device aboard, the bow man will take a fix as they approach within a few kilometers of where they think the FAD is located and will point or shout navigation directions to the captain. Captains will reduce speed as they search. Some FADs have flags to aid the search. Once they have located the head, they will often not travel right to it, rather first trolling the area in order to catch live bait to set on the first traps of the day. Once they have their first bait, the captain will approach the head to check the direction of the attraction to gauge a more precise direction of the current.

The primary technique used in Dominica to catch tuna and marlin around a FAD involves setting what the fishers refer to as traps and results in boat movements consistent with ARS. Similar to the technique involved in float or jug-fishing [45], traps are crafted from meter-long floats (referred to as fenders) that are secured to a length of fishing line (~100m) and a baited hook. The fishers travel up-current from the FAD and throw the free floating trap out of the boat with the goal that it drifts down-current past the FAD head. If a fish does not take the bait, the trap is collected and the process is repeated. Boats usually deploy multiple traps simultaneously and since trap-setting and collecting occur at opposite ends of the patch space, the resulting tracks (discussed below) are characterized by back and forth movements around the FAD head as traps are set and collected. In addition to trap setting, drift fishing is also common around FADs and is characterized by slow speeds where the engine is off or idling and the boat is moving slowly with the current as the fishers use hook and line to catch bait or other smaller fish.

The section above describes search for deep water FADs; other kinds of patches are found other ways. The shallow water FADs referred to locally as the Twins are close to shore and easily found using landmark navigation. Deep water FADs, on contrast, are placed at a distance from shore so that such landmark navigation is usually difficult. A *tash* is a school of fish, most commonly dolphinfish. Fishers are often alerted to *tash* patches by the presence of birds, often frigatebirds (*Fregata* spp.) that are feeding on the same prey, often flying fish, as the target prey fish. During the channel season, in the winter and spring, search focuses on these birds. In the vernacular, *wood* generically refers to floating patches of debris where fish are commonly encountered. Drifting logs, timbers, marine litter such as plastic objects and polystyrene foam, lost fishing gear, and *Sargassum* sp. (a type of seaweed) will also attract fish into patches [46]. Birds are also present at these wood patches.

### Visual analysis

**Fig 2:**
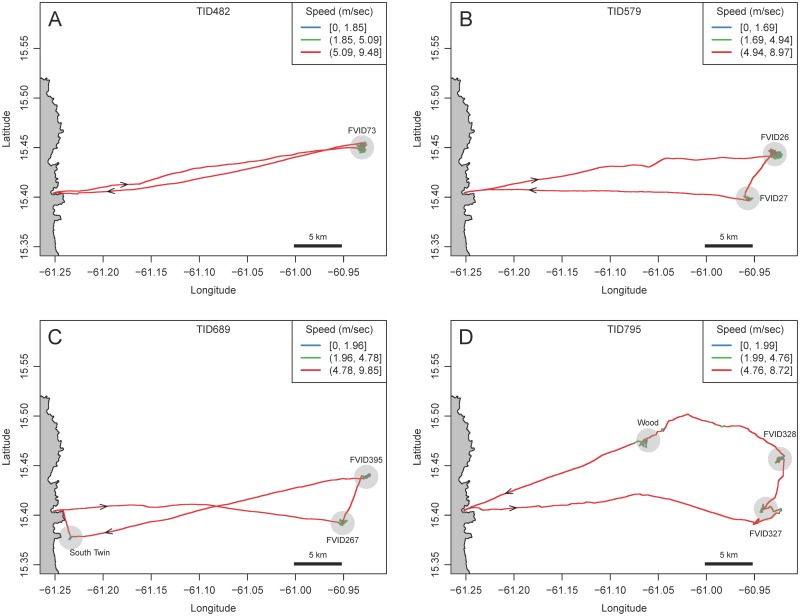
Four example GPS tracks of foraging Dominican FAD fishers. The east coast of Dominica is indicated on the left of each plot. Trips originate at Desa Ikan and travel eastward toward the FADs. Track points are colored to indicate speed. (A) Trip to one FAD. (B) Trip to two FADs (C) Trip to a FAD and a near shore shallow water FAD. (D) Trip to two FADs and a *wood*. Each track is identified by a Trip ID number (TID). Arrows indicate direction of travel. Transparent blue circles indicate areas that were identified as patches by the observer and subsequently identified as ARS. See Fig. 5 for a higher resolution plot of tracks at patches.

We first did a visual analysis of the trips by examining plots of their trajectories mapped onto the seascape. Results show that the captains were practicing classic central place foraging [47]. After departure from Desa Ikan, each boat traveled with ballistic (i.e., straight line) movement directed toward locations where intensive search was subsequently conducted at the FAD patches. The 22 trips analyzed here were sampled for focal follow observation because the captains stated targets were deep water FADs. After foraging at various patches, boats returned to their starting point at Desa Ikan via ballistic travel. Four example tracks plotted in Fig. 2 highlight some of the variability in the nature of the trips.

**Fig 3:**
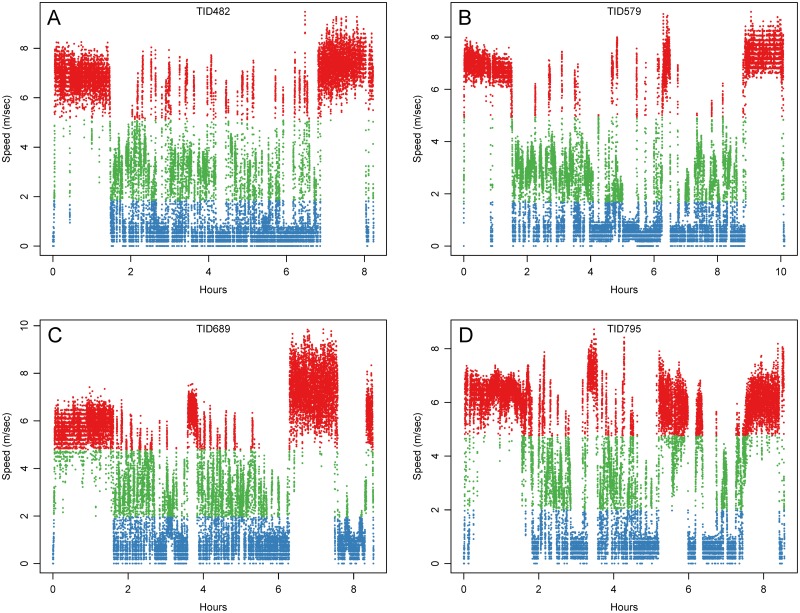
Plot of speed versus time for FAD fishing trips. Plots show the four trips in Fig. 2. Points are color coded in the same way. Periods of travel are fairly homogenous, while behaviors at patches show heterogeneity.

**Fig 4:**
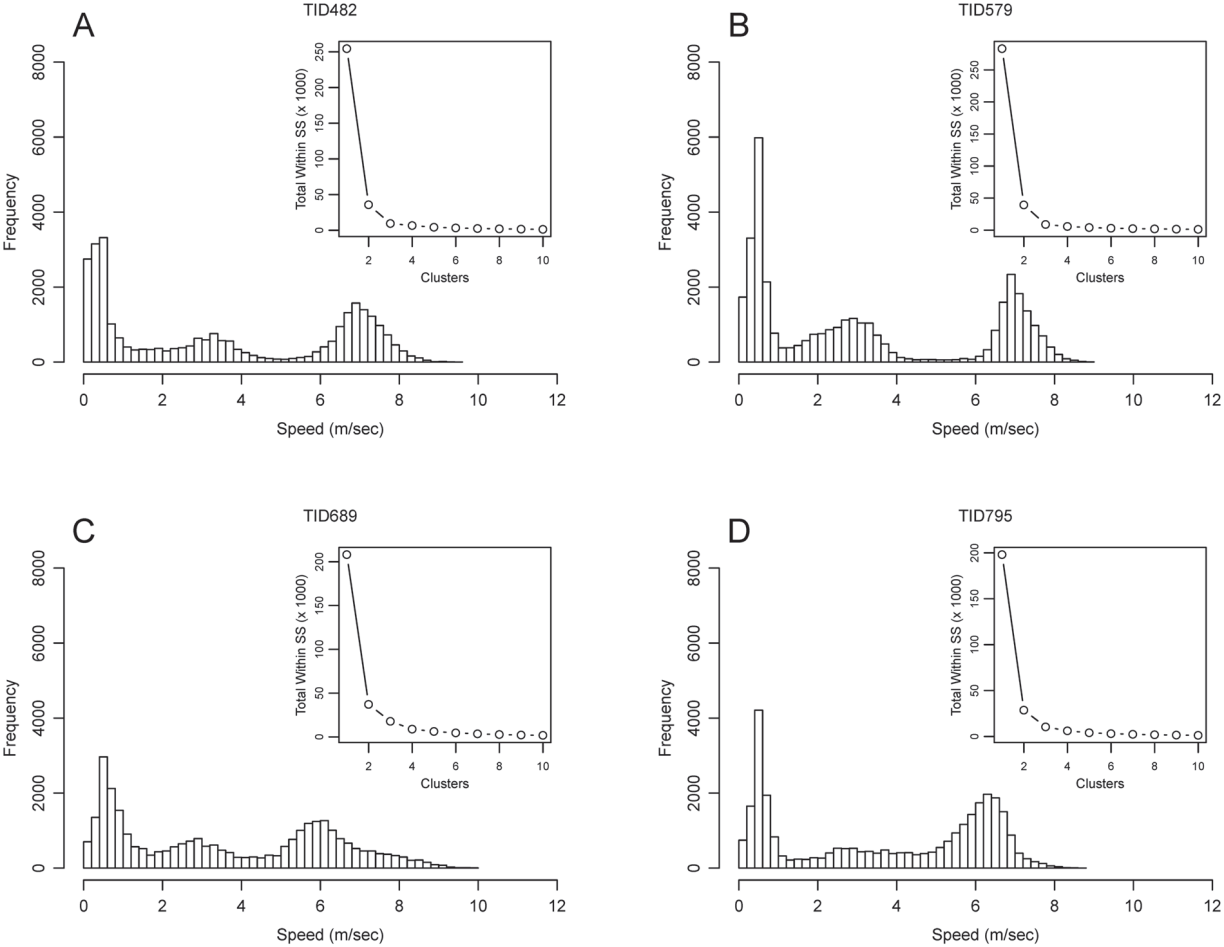
Frequency distribution of speeds. Plots are for the trips in Fig. 2. Histograms show the tri-model pattern characteristic of all the trips in the sample. The inset scree plots show an inflection point at three groups indicating that fewer clusters have a sharply increased total within sum of squares.

Table 1 provides summary data on the N = 22 trips. Crew size was 2 men for each trip (not counting observer). Trip length varied from 6.9–10.9hr with a mean of 8.87hr. We calculated instantaneous travel speeds from the distance traveled between each pair of consecutive one second locations. The mean speed for the trips ranged from 2.97m/s to 4.17 m/s (SD = 2.53–3.57). As mentioned above, one boat and its two captains accounted for 21 of the observed trips. Mean travel speed did not differ between the two captains on the same boat [3.30m/s [n = 5] versus 3.44m/s [n = 16]; t(6.5) = -1.1, p = 0.30]. The one observed trip in the second boat captained by a third man traveled at a mean speed of 4.17 m/s. The small sample precluded testing for differences between the boats. Time series plots of speed for the four example trips shows heterogeneity along the series with periods of consistently higher speeds that observers associated as travel, and periods of slower speeds that are associated with drifting and slow powered movement at patches (Fig. 3). The frequency distributions of travel speeds show a consistent tri-modal distribution. Fig. 4 shows four representative histograms and scree plots. We used k-means cluster analysis to bin and calculate the means for each mode for each trip. The results are presented in Table 2 and show a mean range of 0.37–1.06 m/s for the first mode, 2.84–5.23 m/s for the second, and 6.17–8.54 m/s for the last. Multimodal speed distributions have been identified for nonhuman foragers [48,49], as well as fishers [50–52] and have been used to identify different states of behavior. For example, Walker et al. [52] use Vessel Monitoring System (VMS) GPS data from the French purse-seiner fleet in the Indian Ocean to identify three modes of travel speed that they identified as stop, track, and cruise behavior.

**Table 1:** Parameters for individual foraging trips by Dominican FAD fishers.

TripID	Date	Trip Duration (h)	Distance traveled (km)	Average speed (m/s)	Standard deviation speed	N
474	8/31/2012	8.06	103.71	3.66	2.78	28,586
482	9/1/2012	8.24	101.69	3.48	2.95	29,378
512	9/5/2012	8.80	110.30	3.48	2.78	31,696
519	9/6/2012	9.52	102.11	2.98	2.97	34,271
526	9/8/2012	10.95	124.74	3.16	2.86	39,420
567	9/17/2012	8.60	98.07	3.18	2.89	30,895
571	9/18/2012	9.44	109.78	3.23	2.74	33,968
579	9/19/2012	10.12	115.27	3.16	2.78	36,421
593	9/21/2012	8.67	114.88	3.68	2.70	31,211
596	9/24/2012	6.86	90.53	3.66	2.69	24,722
606	9/26/2012	9.81	110.18	3.12	2.93	35,320
640	9/28/2012	10.27	115.42	3.12	2.77	36,989
649	10/1/2012	7.83	102.94	3.65	2.74	28,192
672	10/5/2012	9.31	111.26	3.32	2.76	33,525
682	10/8/2012	8.99	113.56	3.51	2.78	32,344
689	10/9/2012	8.53	109.80	3.59	2.61	30,647
713	10/16/2012	9.09	112.53	3.44	2.81	32,716
731	10/18/2012	8.06	102.69	3.54	2.59	29,010
757	10/22/2012	8.18	122.98	4.17	3.57	29,472
777	10/24/2012	8.35	111.96	3.72	2.59	30,058
789	10/26/2012	8.95	103.32	3.20	2.71	32,234
795	10/27/2012	8.56	114.70	3.72	2.53	30,812
Mean		8.87	109.20	3.44	2.80	

**Table 2:** Results of k-means analysis of travel speed.

Mode	Range of means (m/s)	Mean of means (m/s)
Mode1	0.37–1.06	0.63
Mode2	2.84–5.23	3.36
Mode3	6.17–8.54	6.82

Mode 1 is associated with drifting with current. Mode 2 is slow powered movement; Mode 3 is full powered travel.

**Fig 5:**
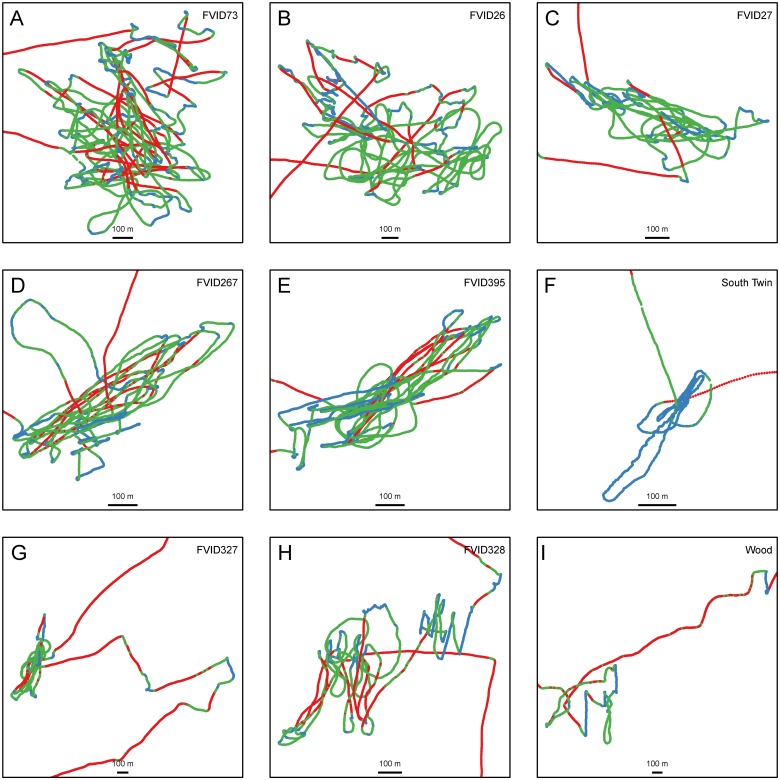
Higher resolution plots of the track segments identified as patches in the tracks of Fig. 2. These segments were identified as patches by the observer and subsequently identified as ARS in the analysis. Tracks are color coded to indicate speed according to the legends in Fig. 2. Within patch movements show speed heterogeneity and patch sinuosity characteristic of ARS.

For our data, the three modal speeds are temporally distributed over the course of the trip in a way that is consistent with gross behavioral differences associated with different speeds noted by the observers. Persistent high speed (mode 3) was associated primarily with travel to patches at the start of a trip, travel between patches during the course of the trip, and return travel to Desa Ikan at the end of the trip. Slower speeds were associated with drifting (mode 1) and slower powered movement (mode 2) in patches; we did experience occasional high speed in patches but it was for short duration. This was confirmed when we examined the tracks visually with the points binned and colored-coded for speed according to the three k-means modes specific to each trip. At a gross visual spatial scale, travel seems to dominate the tracks in Fig. 2, but at higher resolution we see periods of speed heterogeneity, chiefly at the patches. Fig. 5 illustrates, for the example tracks, the segments that we identified as patches based on our observations and subsequently identified as ARS (below). Variance in speeds is clearly apparent with the points color-coded and showing that activities at the patches involve all three modes of speed identified from the k-means analysis. While difficult to discern from a spatial representation, over the course of a trip fishers often allocated more time to mode 1 (i.e., drifting) than to mode 3 (traveling). Pooling all the points across all the trips (N = 701,887, 195.17 hours), find 42.6% are mode 1, 21.4% mode 2 and 36.0% mode 3.

### Segmenting

One of the key methods of movement analysis involves parsing tracks into segments [53] based on spatiotemporal criteria such as location, speed, heading, and sinuosity [54]. A segment is defined as a temporally contiguous portion of the trajectory that is homogenous with respect to criteria chosen to correspond to behaviors of interest. For our purposes, the challenge is to segment the track into two types of behavior—travel and ARS. There are a variety of methods used to identify ARS in foraging animals using GPS track data, including first passage time [55], fractal landscape method [56], multi-scale straightness index [57], net squared displacement [58], behavioral change point analysis [59], and state space models [60].

For our analysis we follow Knell and Codling [29] who developed a method they refer to as the partial sum method. It is based on the cumulative sums of deviations from the mean values of three different movement path properties: speed, absolute turning angle (direction), and the sum of the normalized absolute turning angle and inverse of the normalized speed. We chose a cumulative sum method for its analytic simplicity and demonstrated efficiency correctly categorizing time steps in simulated movement trajectories. Cumulative sum methods are commonly used in quality control applications and are designed to identify prolonged periods of consistent directional deviations from normal [61,62]. We follow their methods below, but limit our analysis to speed for two reasons. First, as we will demonstrate below, speed works very well for our purposes. Using speed alone we are able to identity each ARS segment during all 22 trips without error. Second, results obtained using turning angle produce inconsistent and erratic results. The measure described as “turning angle” by Knell and Codling actually represents the direction of travel during one time period. As discussed in more detail in S1 Appendix, consistent deviations from mean direction do not necessarily indicate ARS.

For each foraging trip in the sample, we calculated the mean speed $\left(\overset{-}{S}\right)$of all steps and then, for each step (*S*_*t*_) in the time series, we calculated the deviation from the mean$\left(S_{t}-\overset{-}{S}\right)$. Deviates greater than the mean speed have positive values and those less than the mean have negative values. We then calculated the cumulative sum of deviations across all steps using the following cumulative sum equation [29]:

$$C_{\tau }=\sum_{t=2}^{\tau }\left(S_{t}-\overset{-}{S}\right)$$ (1)

**Fig 6:**
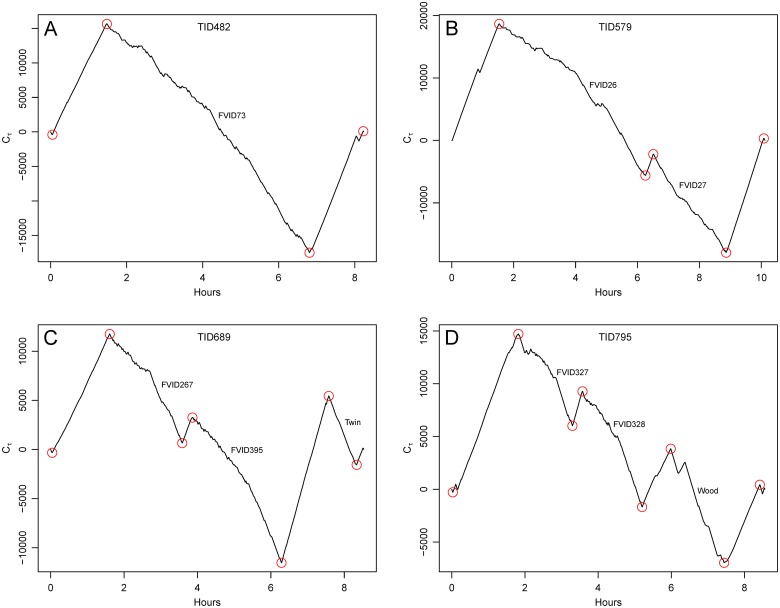
Cumulative sum time series plots generated from Equation 1. Plots are for the trips in Fig. 2. Local maxima and minima are indicated by the red circles, corresponding to points where the track changes between travel and ARS. Segments with positive slope are all travel. Segments with negative slope are ARS and are annotated to indicate patch type identified by observers during trips. FVID indicates a FAD visit identification number.

The cumulative sum plot that results from Equation 1 shows consistent positive slope when the forager consistently travels at speeds greater than the mean; the slope is negative when the forager moves persistently at speeds less than the mean. Fig. 6 shows how the cumulative sum plots for the four trips in Fig. 2 describe behavior during the trips. The time series for trip TID482 (Fig. 6, panel A) shows three major segments (TID refers to the trip identification number). The first segment is a period of consistent positive deviations from the mean trip speed corresponding to observed high speed travel from Desa Ikan; the segment starts at approximately t = 0 and ends at approximately t = 5,000. Next is a period of negative deviations from the mean trip speed that continues until approximately t = 24,000. This segment indicates ARS and will subsequently be associated with a FAD visit (FVID73) to FAD 22 (see below). The third and final segment is a section with positive slope associated with travel back to Desa Ikan that continues until approximately the end of the trip. The cumulative sum plot for TID579 (Fig. 6, panel B) shows two major segments of ARS (negative slope) corresponding to two FADs (FVID26 and FVID27), between which is a period of travel between the patches (negative slope). Panel C is the plot for TID689 and includes two FAD visits (FVID267 and FVID395) and a third negatively sloped segment corresponding to an ARS at a shallow water FAD. Finally, the plot for TID795 shows two FAD visits (FVID327 and FVID328), and an ARS at a *wood* (Fig. 6, panel D).

The analysis above identifies major ARS and travel segments, but a closer examination of the cumulative sum plots identifies other possible candidates for segments that suggest ARS. Within travel segments there are sometimes short periods of slower movement that we do not want to identify as ARS. A captain might slow the boat in order to urinate, to evaluate passing weather, to change the gas line from one tank to the other, to recover from a very large wave, or for a variety of other reasons unrelated to ARS. For example, there is a short period of what appears to be ARS on the second travel segment of TID482 (Fig. 6, panel A). In this case, the boat briefly stopped in response to the presence of another boat whose captain asked for bait. Conversely, movement within a patch might include brief periods of rapid movement from one point within a patch to another during ARS. For example, for TID795 there appears to be a short period of travel within the third period of ARS at the *wood* (Fig. 6, panel D). What counts as a separate bout of ARS? A problem with methods used to examine movement (as well as other ecological data) is that the results are often dependent on the spatial and temporal scale of the analysis [23,63,64]. If the resolution is too fine we run the risk of making a false positive error; that is, we might identify noise as ARS within a period of travel. If the resolution is too course, we risk missing an ARS patch, identifying it instead as travel.

Knell and Codling [29] develop a procedure called the Max—Min Algorithm (MMA) that they designed to identify the cut points along the cumulative sum time series plot where speed changes from segments of above average speed (rising curve) to below average speed (falling curve) and vice versa. The cut points are identified as local minima and maxima and identify the start and endpoints that define segments associated with different behavior. In order to set the appropriate scale, the Max—Min algorithm uses a window or threshold (*w*) which is the timeframe over which a particular maximum or minimum remains so. Once an extremum is tentatively identified, the algorithm continues to look past that point along the cumulative time series to see if the point remains extreme. If another point is identified within the window that is higher than the peak or lower than the valley being considered, a new tentative extremum is identified and the previous discarded. If no new tentative point is found and the length of the window has been exhausted, the original point is identified as a permanent maximum or minimum and the algorithm moves on to search for the next extremum. We coded the algorithm in R [65] (S1 R Script).

Knell and Codling [29] offer a method to determine the optimal size of *w* by first noting that there is an inverse relationship between window size and number of segments produced. They suggest that the optimal window size lies within the range of values that provide the first relatively long consecutive period without a change in the number of segments. We determined the size of the window inductively as the smallest window that identified each patch, including FAD visits, as determined by the focal follow data. The smallest window that identified all the FAD ARS was *w* = 998 and we chose *w* = 1000 for the following analysis.

For the 22 trips, with the window set at *w* = 1,000 seconds, the algorithm produced a total of 178 segments and we assigned each to a behavioral category based on the focal follow observations (Table 3). Travel is defined as sustained, above average speed. Eighty-two of the segments were identified as travel with a mean speed of 6.5m/s; this closely matches the mode 3 speed identified from the k-means analysis (mean mode 3 across all trips is 6.82m/s; Table 2). Based on the criteria of sustained periods of consistent directional negative deviations from normal speed, we initially classified ninety-six of the segments as ARS. These segments had mean speeds ranging from 0.75m/s to 1.76m/s with a mean of 1.32m/s. These are speeds corresponding with mode 1 and 2 identified from the k-means analysis (mean mode 1 and 2 speeds across all trips are 0.63m/s and 3.36 respectively; Table 2).

**Table 3:** Patches and behaviors associated with segments.

Behavior	Number of segments	Total time (hr)	Average speed (m/s)
FAD	42	104.76	1.56
Travel	82	73.83	6.48
Wood	9	8.14	0.97
Twin	6	5.78	0.75
Trip start	20	1.02	1.12
Tash	1	0.77	1.76
Trip end	16	0.38	1.30
Social	1	0.30	1.28
Search	1	0.19	1.54

**Fig 7:**
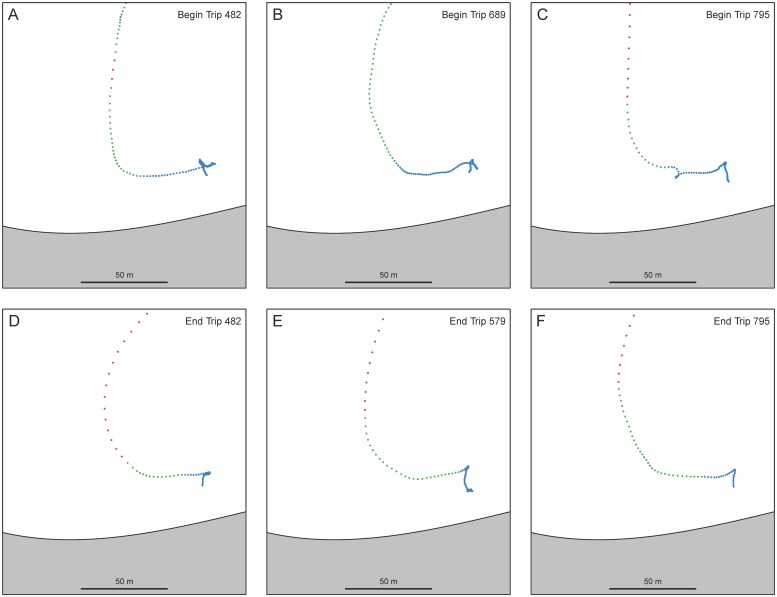
Travel segments in the harbor as trips were getting underway or coming to an end. These segments were misidentified as ARS in the MMA analysis and describe slow movement near the landing site.

Data from the focal follow observations indicate that not all of the segments initially identified as ARS involved intensive search at resource patches. Thirty-six of the segments were short segments at the very beginning (N = 20) or end (N = 16) of a trip with mean lengths of 184 seconds and 85 seconds respectively. Most of these cases involve slower movement at the landing site in the harbor as a trip was getting underway or coming to an end (Fig. 7). Eighteen of the remaining segments identified as ARS were patches that did not involve FAD fishing. Nine of the patches were encounters with floating debris (*wood*). One was an encounter with a school or *tash* of dolphinfish. One segment identified as ARS was a period of socializing with other boats that were met at sea. Six segments were ARS at one of the shallow water FADs (Twins). Finally, one segment of ARS involved an intensive search for the FAD itself that was ultimately not found. FADs are not always found with ease. The remaining 42 segments are associated with the 42 FAD visits identified during observations.

The cumulative sum method combined with the MMA predicts precise times when ARS starts and stops at each FAD providing an objective and reproducible method for delineating patch arrival, leaving, and residence time—measures important for a wide variety of foraging problems [28,66,67]. In order to determine the accuracy of cut points produced by the MMA we compared them to the observations of arrivals and departures from FAD patches we observed during the trips. MMA provides resolution to the second, but the observations were recorded with one minute resolution so we rounded observations by adding 30 seconds. The mean difference between the MMA estimate of the arrival times on the FADs and the observed is about-36 seconds. In other words, on average the method predicts the start of FAD ARS about a half a minute earlier than the focal follow reported. Some of the difference is due to cases where the FAD head was difficult to find after the boat had arrived at its expected vicinity. In this case, the algorithm correctly identifies the beginning of ARS, but it is ARS for the FAD head instead of ARS for fish. A paired-samples t-test shows the difference between observed and expected is not significantly different from zero [t(41) = -0.9337, p = 0.3559]. The mean difference between the observed and MMA estimates of FAD departure is 54 seconds; this means that the algorithm estimates departure from the FAD on average about a minute after the observer indicates departure. This difference is small but statistically significant and probably represents the time it takes to accelerate the boat to traveling speed after the captain indicated to the observer that they were leaving [t(41) = -4.119, p = 0.0001]. Another way to examine the error is with respect to total time in the patch. The average observed patch residence time for the FAD visits is 2.46hrs (8,874 seconds). Errors of 54 and 36 seconds represent 0.6% and 0.4% of this number respectively, a small and probably acceptable degree of error for many applications. The average predicted patch residence time for the FAD visits is 2.49hrs; this differs from observed by 1.3%.

## Discussion and Conclusions

This analysis of GPS tracking data was designed to discern ARS from travel in order to identify when Dominican fishers were exploiting FAD patches. Following the work of Knell and Codling [29] our analysis focused on changes in boat travel speed to identify ARS at FAD patches as periods of consistent negative deviations from the mean trip speed. The cumulative sum method was able to correctly identify all the patches exploited by fishers, including all the visits to deep water FAD for the sample of 22 observed foraging trips. The cut points identifying the beginning and ending of FAD patch residency predicted by the model closely matched the times indicated by direct observation during focal follows.

Foraging theory provided theoretical expectations concerning the nature of fishers’ movements around FAD patches [20,22]. Data from observations of the fishers at FADs used in conjunction with simultaneously collected GPS tracking data were used to validate our understanding of ARS behavior at FAD patches in terms of movement and we confirmed the theoretical expectations. At FAD patches, boats slow to a quarter of travel speed (Table 3). Speed is also more variable within patches (Fig. 5) with ranges across all three modes identified earlier (Fig. 4). Movement is also clearly more sinuous at FAD patches but that was not tested in this application (see more on sinuosity below).

The methods we use here are similar to Vessel Monitoring Systems (VMS) used by some nations to monitor fishing effort in managed areas while avoiding the cost of placing monitors on all the vessels [51,68]. These methods use location data, often collected via GPS, ARGOS, and Inmarsat systems to identity periods of fishing and non-fishing from the spatial data. Validation with onboard observers for a sample of trips is key. Chang and Yuan [69] report that vessel speed is the most widely used criteria to characterize fishing activities. See also Table 1 in Lee et al. [51]. The data used in these systems often have coarse resolution and simply use a speed category to classify a particular fishing activity.

Characterizing fishing effort across space is both challenging and crucial for both evaluating the sustainability and environmental impacts of both industrial fishing and small scale artisanal fishing [70]. Spatial analysis to understand effort by artisanal fishing is limited (see however [71–74]) but takes on new urgency as fishers adopt more effective fishing gear such as FADs. High quality information concerning how fishers use fish aggregating devices is important for setting policy and management goals with regard to fish aggregating devices and their impact on fisheries in the Caribbean and other regions [10,75,76].

Confidence in results from GPS data is important because while direct observation of foraging is ideal, it is difficult—often impossible, time consuming, and costly. In our case, having observers aboard a boat is inconvenient for fishers and involves risk to the observers. A large sample requires many observers. In contrast, for the application here, the advantages of the validated GPS method include a larger sample, reduced risk and cost, computational and analytic simplicity, and very good results. Our results provides a level of confidence and caution as we plan to apply the method to the larger sample of foraging tracks for which we have not collected focal follow data.

The small sample of observed boats and captains does raise the issue of whether the cumulative sum method we describe in this paper is applicable to other captains and boats in our larger sample of trips at Desa Ikan. There is good reason to believe that the relatively small sample of observations provides a sufficiently accurate description of FAD trips to generalize the method to other boats and captains. Visual analysis of tracks from the larger sample of FAD trips indicates a general pattern of directed, higher speed travel between patches and slower and sinuous movement at FAD locations and other presumed patches [33]. Discussions with fishers confirm that the pattern of movement at FADs identified during the observed trips as ARS is a general feature of FAD fishing at Desa Ikan. Foraging theory predicts foragers should adopt ARS at patches [20,27] and the evidence supports the conclusion that FAD fishermen at Desa Ikan do. At Desa Ikan, different boats and captains may travel at slightly different speeds during trips, but it should be kept in the mind that the cumulative sum method scales to the mean speed of each particular trip and not the mean speed of all trips.

Prudence should also be exercised moving forward with respect to generalizing the model across different fishing or foraging contexts. The trips we included in this analysis were chosen for focal follows because the captains indicated that FADs were the trip’s intended goal. This created a homogenous sample that well served the goals of the research, but does not represent the breadth of foraging trip types that originate at Desa Ikan. As mentioned above, FAD fishing is one foraging tactic of a suite utilized by the fishers. Characterizing patches encountered during channel fishing and bank fishing trips might be challenging for the methods used here. While our application used only speed, other foraging situations can be imagined where sinuosity would be more helpful; for example in a context where there is less variation in speed between travel and speed while foraging in patch. Similarly, Knell and Coding [29] point out that in situations where travel is a smaller portion of the foraging trip the method becomes less useful. An example of this type of case would be bank fishing for demersals at Desa Ikan. Bank patches are found on the insular shelf close to shore (<10km). Compared to FAD trips, travel is a much smaller portion of the foraging day for these sorts of trips and the mean overall trip speed is lower. This could make it more difficult to distinguish travel from ARS. The temporal length of ARS bouts with respect to the duration of travel segments should also be considered when choosing the threshold used by the Max—Min algorithm to determine what scale of deviation can be ignored as noise and what can be understood to be actual behavioral change.

**Fig 8:**
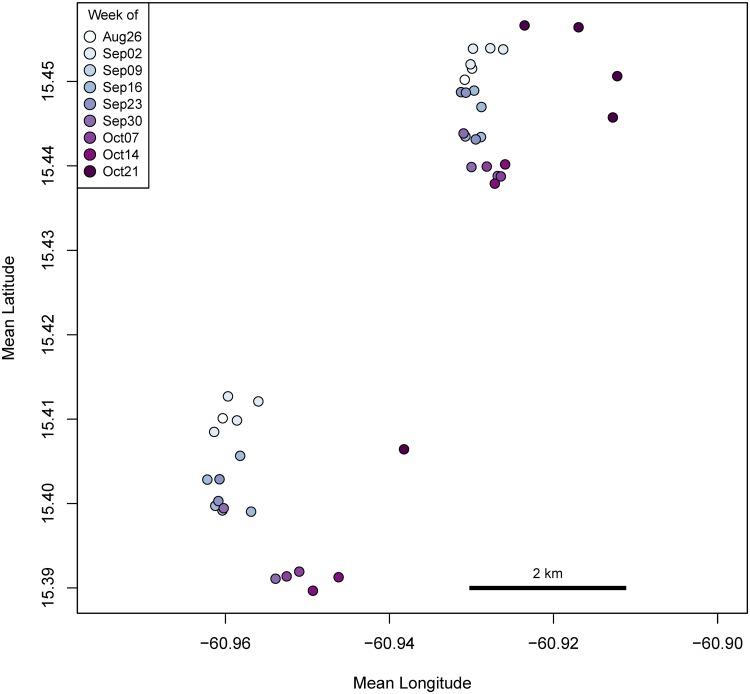
Mean locations of ARS at FADs. The FAD patches were moving synchronously across the seascape, presumably with currents. Points are color coded to indicate the week. The single visit to a third FAD is not shown.

As we apply this method to the larger sample, one challenge will be to distinguish ARS at FADs from ARS at other patches. Since moored FADs are anchored to the ocean floor we expect their associated patches to be found at known, fixed locations to which fishers regularly return over the course of multiple foraging trips. This is not expected for *wood* and *tash* patches. The extent to which FADs are found at fixed locations over time can be seen by examining the location of FAD patches over the course of the sample period. Three different FADs were visited (Table 4). Two FADs received all but one of the visits; the third FAD was newly deployed during the sample period (there were also other additional FADs visited by boats from Desa Ikan during this period). The mean centroid distance to the FAD patches from the central place ranged from 35km-38km. In order to learn if this changed over time, we plotted the mean locations of each FAD patch as determined by ARS to show that the patches were moving in wide arcs over time presumably with the currents (Fig. 8). Recall that during placement, the fishers add scope to the mainline to avoid excess strain and reduce failure. As a result, the patches created by the moored FADs move over the course of weeks and months. To estimate the size of this larger area, we used the maximum distance between two patch locations for each FAD to determine that the area of the drift zones range between approximately 4km^2^ and 5km^2^. For our analysis of the sample of trips without observers, we can confidently identify ARS within these areas as FAD fishing. In addition to movement of the FAD head, fishers report that current can also drag FAD anchors along the ocean floor, sometimes for kilometers. There was no evidence that this occurred during the sample period.

**Table 4:** FADs visited.

FAD	Number of visits	Mean distance from Central place (km)	Mean patch residence time (hr)
19	18	37.9	1.72
22	23	35.2	3.18
22S	1	36.5	0.41

The segments at the start and end trips can be rejected as deep water FAD patches as can the ARS at the sites identified as the Twins which are located approximately 2km from shore. Both *tash* and *wood* patches are also straightforwardly distinguished from FADs. While FADs are essentially stationary over the course of one visit, and are repeatedly found in the same general location over many trips, each *wood* and *tash* patch is independent of others and are encountered at different locations throughout the seascape on different days. In addition, *wood* are free to drift with the current while *tashes* move as a school of fish moves. Visual examination of GPS tracks collected in 2013 during the channel season when fishers were focused on hunting schools of dolphinfish, show *tash* encounters playing out across a swath of seascape larger than FAD patches as fishers search for, follow, and engage schools.

FADs regularly fail in the harsh conditions of the Atlantic and new ones are deployed [77]. For example, in Desa Ikan fishers report that the three FADs identified in this analysis (as well as others) were destroyed in April 2013 by a particular strong tropical easterly wave. In the absence of additional direct observations, we can determine the location of new FAD placements using only GPS data by identifying ARS consistently in the same 4km^2^–5km^2^ area over the course of a number of foraging trips. Our conclusions can be confirmed by fishers’ reports. It is clear that distinguishing FAD ARS from among the other ARS segments benefits from domain knowledge of the system; that is, the analysis can be enhanced by the expert knowledge of the fishers [78]. These methods presented here could potentially be adapted to examine within patch behavior. Fig. 5 shows considerable variation in speed and sinuosity around FADs and presumably reflect the range of different behaviors occurring when a FAD patch is exploited. Some of these behaviors can be discerned visually. For example, drift behaviors can be identified in the tracks as repeated, straight, often parallel, and slow segments across the proscribed area (these segments are colored blue in the Fig. 5). These segments are parallel because they are following the surface current and wind which often tends to remain constant over any particular FAD visit. It might also be possible to distinguish individual trap deployments which would be useful as a measure of how different fishers exploit different FADs.

The capacity to identify patches and infer foraging behavior using high resolution tracking data like those described here is being used to characterize foraging behavior among a wide variety of organisms, especially birds and marine mammals. Those studies usually do not have the advantage of a sample of observed foraging trips and must infer behavior from theory and the tracks alone [25,49,79]. Using direct observation, we were able to associate foraging with ARS at patches; this result can provide researchers who lack observational data with more confidence as they infer foraging behavior based on GPS tracks alone.

The ability to identify patches is important for testing a wide variety of basic foraging models [80–83]. Research with terrestrial human hunters have been successful testing prey choice models [84,85] but have had less success with patch models since patches are difficult to characterize [86]. High resolution spatial data in combination with more sophisticated models to identify resources patches and foraging behavior promise new insight into how foragers move across the landscape (and seascape) and use resources [58,87]. In spite of challenges, the method described here is simple, cost-effective and has good potential for future research.

## Accession Number

The GPS data files are available at the following site: http://repository.tamu.edu/handle/1969.1/152005.

## Supporting Information

S1 Appendix Consistent deviations from mean direction do not necessarily indicate ARS. (DOCX)

S1 R Script Max—Min Algorithm. (TXT)
