# Supplementary material for: Using a Partial Sum Method and GPS Tracking Data to Identify Area Restricted Search by Artisanal Fishers at Moored Fish Aggregating Devices in the Commonwealth of Dominica
Source: PLoS One. 2015 Feb 3;10(2):e0115552. doi: 10.1371/journal.pone.0115552 (PMC4315603; doi:10.1371/journal.pone.0115552)

**Appendix S1**

Knell and Codling’s [29] method is based on cumulative sums of deviations from the mean values of three different movement path properties: speed (their equation 6), absolute turning angle (their equation 7), and the sum of the normalized absolute turning angle and inverse of the normalized speed (their equation 7). While speed works well for our application, the other two movement path properties do not.

It is more difficult to use the cumulative sum approach in general with directional data because it is two dimensional and lacks a meaningful 0 value. For directional data, a 0 indicates a direction of north (for geographic directions measured in degrees) or east (for trigonometric directions measured in radians). Knell and Codling [29] take the approach of taking the absolute value of the arc tangent of the direction of travel in radians (their equation 7). This measure is described as “turning angle” by Knell and Codling, but it actually represents the direction of travel during one time period (one second in our case). This has the effect of wrapping the directions into a quarter circle with 0 representing east and west directions and 1.57 representing north and south directions. Northwest, southwest, northeast, and southeast directions all map to 0.79. Periods of no movement have no defined direction and must be represented as missing values.

In addition to this issue, the interpretation of this measure is more difficult than for speed. This is because greater than average direction indicates travel closer to a N/S orientation and less than average direction indicates travel closer to an E/W orientation. For our data, travel to and from the FADs is approximately E/W so travel is represented as a negative slope on the cumulative curve. ARS is characterized by greater variability in direction (E/W and N/S travel) so these sections have increasing slopes on the plot. One problem is that consistent N/S travel is interpreted as ARS because it is consistent deviation from the mean direction. Travel between FADs which lie at a general N/S orientation to one another is correctly identified by consistent speed greater than the mean, but is *not* identified as travel using direction. This is apparent from a visual examination of the cumulative sum graphs (Figure S1) which compare cumulative sum plots for the three different movement path properties for the four example tracks presented in the main text. Black is speed, red is turning angle, green is the composite property. In the plots, direction and turning angle have been reflected (multiplied by -1) so that increasing values reflect travel and decreasing values reflect ARS. The composite measure was reflected only for TID795. In the other three trips, the composite measure was positively correlated with speed so it was not reflected.

As discussed in the paper, the cumulative sum plot using speed alone correctly identifies periods of travel as consistent positive slope and ARS as consistent negative slope. The cumulative sum plot using direction correctly identifies travel out to and return from the FADs, but misses segments of travel that involve N/S travel between FADs. This can be seen by examining the plots for trips TID579, TID689 and TID795. Each of these trips includes travel between two FADs correctly identified as the short section of positive slope on the black speed plot. For TID579, this travel between FADs occurs at approximately hour 6.5; for TID689 and TID795 it occurs at approximately hour 3.5 (TID482 did not involve any N/S travel between FADs). In each case, the cumulative sum plot using the direction measure incorrectly identifies the travel between FADs as ARS because it is consistent movement in a direction away from the mean direction. This may not have been an issue with the simulated data used by Knell and Codling’s [29] because direction of travel was randomly generated rather than directed as in our case.

Finally, the composite measure (the sum of the normalized absolute turning angle and inverse of the normalized speed) behaves erratically because the reciprocal of speed near the mean speed become very large as the denominator approaches zero. This is apparent from the erratic behavior of the green line in the plot.

**Figure S1.** Cumulative sum time series plots generated from speed (black line), direction (red line), and the composite measure combining speed and direction (green line).


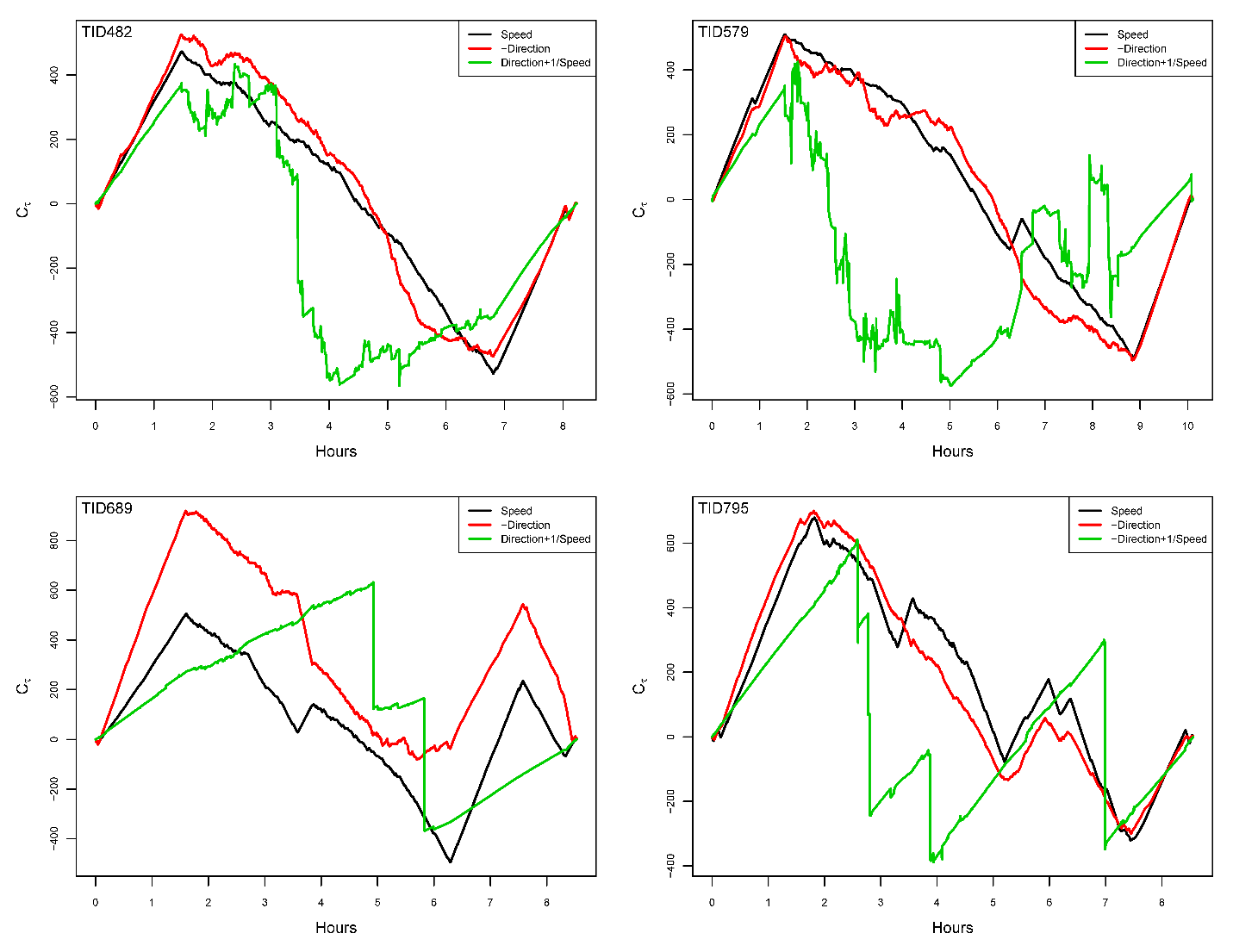

Supplement: S1 Appendix — (DOCX) [file pone.0115552.s001.docx]
